# A Rasch Analysis of the School-Related Well-Being (SRW) Scale: Measuring Well-Being in the Transition from Primary to Secondary School

**DOI:** 10.3390/ijerph18010023

**Published:** 2020-12-22

**Authors:** Daniela Raccanello, Giada Vicentini, Elena Trifiletti, Roberto Burro

**Affiliations:** Department of Human Sciences, University of Verona, 37129 Verona, Italy; giada.vicentini@univr.it (G.V.); elena.trifiletti@univr.it (E.T.); roberto.burro@univr.it (R.B.)

**Keywords:** well-being, achievement emotions, primary school, secondary school, factor analysis, Rasch analysis

## Abstract

Within educational systems, promoting well-being is an essential objective along with traditional aims focused on students’ learning. However, scarce attention has been devoted to school-related well-being in the transition from primary to lower secondary school, also for the paucity of brief instruments deputed to measure it. We assessed well-being at school for fourth-graders and seventh-graders, by adapting and validating the Italian version of the School-Related Well-Being (SRW) scale, using in sequence exploratory factor analysis (EFA), confirmatory factor analysis (CFA), and Rasch analysis. Through the Rasch analysis, we transformed the SRW scale into an instrument that respects the properties of the fundamental measurement. We measured well-being and achievement emotions at time 1 and grades at time 2. The SRW scale correlated with another measure of well-being and with students’ achievement emotions. Grade-level differences emerged, with a decrease of well-being that attested a maladaptive trend at increasing age; moreover, females reported higher well-being than males. Well-being at school was positively linked to achievement. Beyond its methodological relevance, this study highlights the need for developing interventions to support students in the transition from primary to lower secondary school, which is such a pivotal time in their learning path.

## 1. Introduction

Within educational systems, promoting well-being is nowadays an essential objective along with traditional aims focused on students’ learning, and it could also be considered as an indicator of students’ mental health [1,2,3]. According to the perspective of positive psychology, studying subjective well-being and the factors underlying its changes is of key relevance for favoring a good quality of life [4]. However, there is still a lack of consistency in the conceptualization of subjective well-being across different life domains. In addition, notwithstanding documented variations in subjective well-being in different contexts, not much attention has been paid at assessing it using measures pertaining specifically to school [5]. School is the context in which children and adolescents pass almost one third of their waking time, and as a consequence it plays a privileged role for impacting their overall well-being: It is therefore a key environment for positive cognitive, emotional, and relational experiences, but also for negative ones such as academic failure, school anxiety, or bullying [6].

In line with Hascher ([7], p. 99), we could state that research on subjective well-being at school has aligned with at least three different conceptualizations, conceiving it as a “*specific emotional quality of feeling well*”, as a “*supra-term for positive emotions like enjoyment, pride, satisfaction, etc*.”, and as “*a multidimensional concept combining cognitive and emotional factors*”. Notwithstanding differences between the three approaches, they basically agree in identifying enjoyment and/or happiness as a central characteristic of the construct. Coherently with previous works [8,9], we define well-being at school as the psychological condition in which there is a prevalence of positive cognitions and emotions over negative cognitions and emotions, in the life of the members of the school context [10]. This is also consistent with the definition of the World Health Organization [11], according to which psychological well-being is related to a large variety of individual factors pertaining to the cognitive, emotional, and bodily domain. Furthermore, this is in line with the spontaneous conceptualization of well-being as possessed by school children, for whom the salience of the psychological and the physical domain of “feeling well” is the same, as emerged through the analysis of narratives of personal events [12].

Notwithstanding its relevance, scarce attention has been devoted to school-related well-being in the transition from primary to lower secondary school, also for the paucity of brief instruments deputed to measure it. Therefore, the main aim of this work was to assess well-being at school for fourth-graders and seventh-graders, by adapting and validating the Italian version of the School-Related Well-Being (SRW) scale [13]. The SRW scale has the great advantage to be very short, and therefore it can be administered easily. This is particularly advantageous taking into account some constraints typical of surveys with students, and specifically with younger students, such as low attention, necessity to gather data on well-being together with other measures in long surveys, or school organizational time constraints.

### 1.1. Well-Being in the Transition from Primary School to Secondary School and Gender Differences

The transition from primary school to secondary school is characterized by a variety of changes relating to the psychological development, regarding the biological, cognitive, emotional, social, or identity formation functioning [1,14]. Also, contextual changes are very frequent, such as those relating to the school and its organization, to teachers, or to the composition of the class. Beyond changes in these domains, there are variations for learning-related motivation and emotions, both for male and female students. Overall, these changes partially suggest the existence of a maladaptive trend in the transition from primary to secondary school also for students’ well-being, and specifically for constructs such as satisfaction, affect, achievement emotions, i.e., those emotions pertaining to learning activities or outcomes [15,16,17,18,19,20,21], similarly to what happens for motivational constructs such as achievement goals, self-efficacy, or academic self-concept [22].

A few studies also examined gender differences regarding well-being at school, but the findings are not always consistent [15,23]. The literature on this issue is not conclusive: Coherently with results concerning the motivational domain, gender differences for well-being are usually different for different school domains and sometimes they concern only some components of school-related well-being [24,25,26].

Identifying age and gender variations in psychological well-being at school is a key requirement for the development of effective interventions in the schools, providing basic indications for accurate targeting strategies.

### 1.2. School-Related Well-Being and Educational Outcomes

Previous research partially examined the relation between school-related well-being and other educational outcomes. A few studies considered subjective well-being and school achievement, indicating positive associations between the two variables, mostly through cross-sectional designs [27,28,29]. Similar associations were identified also through some longitudinal studies [5,13,30,31,32].

Only some studies examined the relation between subjective well-being or school well-being and achievement emotions. Notwithstanding the large number of studies on test anxiety, research focusing on its relation with subjective well-being is scarce [30,33]. The studies which examined the relations between well-being at school and achievement emotions [5,13,32] revealed that school-related well-being was positively associated with pleasant emotions such as enjoyment and pride, while it was negatively associated with unpleasant emotions such as anxiety, anger, shame, boredom, and hopelessness.

Therefore, given the paucity of studies on these relations, further research is needed to increase knowledge on educational outcomes of school-related well-being in the transition between different school levels, as a basis for successful interventions aiming at promoting students’ well-being.

### 1.3. Measurement of Well-Being at School

Together with a lack of consistency in the conceptualization of well-being at school, current literature is characterized also by a lack of consistency in measurement [5]. From a methodological perspective, there is a need for developing instruments to assess well-being in reasonably short assessment sessions, given both cognitive (e.g., attentional, metacognitive) and organizational (e.g., reduced time) constraints typical of research in the schools.

To monitor changes in students’ school-related well-being, it is of key relevance that measurement instruments are reliable and valid. In the literature, instruments such as the Student Well-Being Questionnaire (SWQ) [10] enable to conduct a valid multi-dimensional assessment of school-related well-being, distinguishing a variety of positive and negative components. However, in some contexts both time and other resources that can be dedicated to monitoring well-being are particularly reduced, as when it has to be assessed with students within school. Taking this into account, short instruments that can be administered rapidly assume a great relevance. An example of such short questionnaires is the SRW scale, a six-item instrument designed to assess students’ overall judgements on their well-being at school [5,13,32]. Previous data demonstrated the internal validity of the scale through confirmatory factor analyses which supported its one-factor structure. Further analyses indicated the good internal consistency of the scale and its discriminatory validity, examining its correlations with prior achievement, self-concept, and achievement emotions.

Possible disadvantages due to the brevity of the instruments can be compensated using the Rasch analysis, which goes beyond the weak points of traditional approaches by giving priority to the objective measurement of the latent dimensions based on the principles of fundamental measurement [34,35,36]. While such principles are respected in the fields of physical and natural sciences, this does not generally happen within the psychological field. Nevertheless, applying the Rasch analysis gives the possibility to build instruments that take them into account, permitting a more accurate quantification of the psychological dimensions which characterize individuals. The Rasch analysis enables to test statistically whether the data fit the requirements and assumptions of a mathematical model whose name derives from the name of its developer, Georg Rasch [37]. Such model is based on a latent trait approach and it is characterized by probabilistic conjoint additivity [38,39]. “Conjoint” refers to the possibility to measure both persons and items on the same scale, while “additivity” refers to the equal-interval properties of the resulting scale.

When the fit of the data to the Rasch model is adequate, it is legitimate both to sum the scores of the items in order to obtain a global score and to transform the instrument into an interval scale, for which the measurement unit is constituted by the logit [36]. Performing a Rasch analysis, different indicators of the quality of the measurement are evaluated. A first step is checking the internal construct validity, through the exam of some general assumptions concerning the item response theory models [40,41,42], i.e., monotonicity [43,44], local independence [45,46,47], unidimensionality [48,49], and absence of differential item functioning (DIF) or item bias [50,51]. The DIF is related to another key assumption of the Rasch model, relating to the fact that the item parameter estimates should not depend on the person sample, and vice versa. This assumption may be violated if certain items are easier or more difficult for some groups of participants–independently from their ability: In this case, we refer to DIF. A second step is to examine reliability, assessed through the calculation of indexes such as the person separation index (PSI) or the Cronbach’s alpha [52,53,54]. A third step refers to targeting, which indicates how well the range of measurement of the instrument corresponds to the distribution of the calibrating sample, using for example person-item maps [34,35].

After conducting these steps, the data could still not meet the assumptions of the model or not fit the Rasch model. In these cases, the scale could be modified, usually through an iterative phase which includes some changes pertaining to the items, in order to reach a solution coherent with the assumptions of the Rasch model [55,56]. Some modification strategies are for example: item rescoring, to account for violations of the monotonicity; item grouping or “testlets” creation, to account for violations of local independence; item splitting, to account for uniform DIF; item deleting, in cases of failures using the other strategies. After the application of each strategy, the fit between the data and the model is verified again. When a final solution which fits the model is reached, the iterative process is interrupted. Given that the global score is characterized by statistical properties such as specific objectivity and sufficiency, the scale can finally be transformed into an interval level measure [36,38].

### 1.4. The Present Study

The general aim of this research was to adapt the SRW scale [13] to the Italian context applying the Rasch analysis, in order to assess school-related well-being in the transition from primary to lower secondary school. We used the scale as a self-report instrument with a convenience sample of fourth-graders and seventh-graders. Documenting how school-related well-being varies is essential to develop intervention programs to support students’ well-being in this delicate step of their school path. Compared to previous works, the added value of this study is to use Rasch modeling to construct an instrument that measures school-related well-being which respects the properties of fundamental measurement [35,36], focusing on the transition from primary to lower secondary school. We had four main aims.

The first aim was to develop an instrument assessing school-related well-being which respects the properties of fundamental measurement through the Rasch analysis, by adapting the SRW scale to the Italian context [13]. A preliminary step was to establish the internal validity of the scale, examining its factorial structure: We expected the scale to be unidimensional (*Hypothesis 1a*). We then could transform the scale applying a Rasch analysis, further exploring the unidimensionality of the scale. We also expected a uniform functioning of the scale according to class level and gender (*Hypothesis 1b*).

The second aim was to investigate the external validity of the SRW, examining its linkages with both another questionnaire designed to assess school-related well-being, the SWQ [10], and students’ judgments on their school-related achievement emotions. We expected students’ scores on the SRW scale to be positively related with the three positive dimensions of the SWQ, i.e., positive attitudes towards school, enjoyment in school, and positive academic self-concept; and to be negatively related with the three negative dimensions, i.e., worries in school, physical complaints in school, and social problems in school (*Hypothesis 2a*). Moreover, we expected students’ evaluations of the SRW scale to be related positively with positive achievement emotions (enjoyment, pride, hope, relief, relaxation) and negatively with negative achievement emotions (anxiety, anger, shame, boredom, hopelessness) (*Hypothesis 2b*).

The third aim was to examine whether school-related well-being varied according to class level and gender. We hypothesized a decrease of school-related well-being at increasing ages (*Hypothesis 3a*). We did not formulate any specific hypothesis for gender.

The fourth aim was to study the relations between school-related well-being and later achievement. We hypothesized school-related well-being to be positively related to grades in native language and mathematics (*Hypothesis 4a*).

## 2. Materials and Methods

### 2.1. Participants

We involved a convenience sample, which consisted of 432 students attending the fourth (*n* = 256; *M_age_* = 9.54; *SD* = 0.33; 45% female) and the seventh grade (*n* = 176; *M_age_* = 12.50; *SD* = 0.34; 50% female) in 19 primary and lower secondary schools which were part of four school institutes in Northern Italy. Students’ families were characterized by a wide range of socio-economic backgrounds. The students participated after their parents had signed the consent form. The inclusion criteria were (a) attending the fourth or the seventh grade in the involved schools, and (b) having the linguistic and cognitive abilities necessary to take part to the proposed activities.

This research is part of the “School and social cohesion in Italy” project [57], supported by the Department of Human Sciences of the University of Verona as an Interdisciplinary Research Action. The project received the approval of the Local Ethical Committee of the Department of Human Sciences of the University of Verona (protocol number 362742).

### 2.2. Procedure

We met the principals and the teachers of the involved schools to present the research project, and then we sent the informed consent forms home to parents. We sent 873 invitations, including all the students of the involved school classes for which we had obtained the permission by the principals and the teachers. The choice of the school level was related to the need to examine the central classes for both primary school and lower secondary school. The study was voluntary; therefore, the parents could freely decide not to give their authorization. For ethical reasons, the students who had linguistic or cognitive impairments and had the permission to participate completed the questionnaire, but their data were excluded. The students whose parents agreed for participation filled in the questionnaires in class sessions lead by two investigators. The students who did not have the permission to participate to the research were involved in alternative school activities guided by their teachers.

We collected the data with the students over two waves during the school year 2018–2019. The first wave was in the middle of the first term (time 1), while the second wave was at the end of the first term (time 2). At time 1, we assessed students’ school-related well-being and achievement emotions, focused on classes or tests. The students were told that all the items would have been read aloud, that there were no right or wrong responses, to answer sincerely, and that the questionnaires were anonymous. There was a familiarization phase, including examples on how to answer. At time 2, we obtained the grades in native language and mathematics from the schools. All the verbal labels used in the instruments were appropriate to students’ gender, with male and female versions, to favor their identification. At the end, we informed both teachers and parents on the findings of the research.

#### 2.2.1. Well-Being at School

We assessed students’ well-being at school through two scales, the SRW scale [13] and the SWQ [10]. We developed an Italian version of the SRW scale [13] by means of a back-translation procedure, from English to Italian and back from Italian to English. The SRW is a 6-item unidimensional scale assessing well-being at school (see Table 1 for the English and the Italian versions). The instruction used in this study were: “The following sentences refer to how you feel at school. For each sentence, put a cross on the number which corresponds to how much the sentence is true for you. You can choose among the following alternatives: 1 = *not at all*, 2 = *slightly*, 3 = *moderately*, 4 = *very*, and 5 = *extremely*”. The students evaluated how much each item was true for them (e.g., *I feel good at school*) on a 5-point scale (1 = *not at all* and 5 = *extremely*).

We administered also the SWQ [10]. It is a 19-item scale on well-being at school which comprises six dimensions, three positive and three negative: positive attitudes towards school (e.g., *Whatever happens, the school has something good*), enjoyment in school (e.g., *It happened that I was happy at school because I did something that I like*), positive academic self-concept (e.g., *I don’t have problems mastering school tasks*), worries in school (e.g., *I was worried because of the school*), physical complaints in school (e.g., *It happened that I had stomachache because of the school*), and social problems in school (e.g., *It happened that I had problems with my classmates*). The students evaluated how much each item was true for them on a 5-point scale (1 = *not at all* and 5 = *extremely*).

#### 2.2.2. Achievement Emotions

To assess school-related achievement emotions, we used a brief version of the Achievement Emotions Adjective List, AEAL [19,58,59,60,61,62,63,64,65]. The AEAL is a 30-item self-report questionnaire measuring ten achievement emotions: three positive activating emotions (enjoyment, pride, hope), two positive deactivating emotions (relief, relaxation), three negative activating emotions (anxiety, anger, shame), and two negative deactivating emotions (boredom, hopelessness). To avoid students’ cognitive overload due to the length of the questionnaire, we used a reduced version of the AEAL comprising ten adjectives, one for each achievement emotion. We had already used this version with students of the same age [19,64,65]. We administered the AEAL twice, separately for two settings, i.e., lessons and tests (oral or written evaluations). The students evaluated how much intense each emotion was (i.e., *happy, proud, hopeful, relieved, calm, anxious, angry, embarrassed, bored, sad*) on a 5-point scale (1 = *not at all* and 5 = *extremely*). We then calculated two composite scores, relating to positive and negative achievement emotions.

#### 2.2.3. Grades

We obtained from the schools the students’ grades separately for native language and mathematics as included in their final report card, assigned at the end of the first term of the school year. In the Italian education system, grades range from 1 (very poor) to 10 (excellent).

### 2.3. Data Analysis

We used the R statistical environment for all analyses, Version 4.0.3 (R Foundation for Statistical Computing, Wien, Austria) [66]. Concerning the first aim, to examine the internal structure of the SRW, we ran a parallel analysis, a very simple structure analysis, an exploratory factor analysis (EFA), and a confirmatory factor analysis (CFA). Through preliminary analyses (i.e., Bartlett’s test of sphericity; Kaiser-Meyer-Olkin, KMO), we checked whether our data were suitable for factor analyses. The KMO measure of sampling adequacy tests whether the partial correlations among the variables are small. Bartlett’s test of sphericity verifies whether the correlation matrix is an identity matrix, which indicates that the factor model is inappropriate. The parallel analysis and the very simple structure analysis are two methods which enable to identify the appropriate number of factors within an EFA [67]. The parallel analysis compares the eigenvalues related to the random data, derived from the original database, with the eigenvalues related to the factor analysis, in order to determine whether the factors are mostly random noise. The very simple structure criterion allows to compare solutions of varying complexity and including different numbers of factors. The graphic output indicates the optimal number of factors for different levels of complexity. 

We conducted an EFA taking into account as fit indexes the Tucker-Lewis index (TLI), the root-mean-square error of approximation (RMSEA), and the standardized root mean residual (SRMR), with TLI ≥ 0.95, RMSEA ≤ 0.08, and SRMR ≤ 0.06 as threshold values [68]. We then ran a CFA, with the maximum likelihood with robust Huber-White standard errors and scaled test statistic (MLR). For running a CFA, the minimum ratio between the number of observations and the number of parameters should be 5:1 or more, and preferably 10:1 [68]. In our case, we had 12 estimated parameters with 432 participants, therefore the ratio was 36:1, and the sample size resulted adequate. We considered as fit indexes the comparative fit index (CFI), the TLI, the RMSEA, and the SRMR, with CFI and TLI ≥ 0.95, RMSEA ≤ 0.08, and SRMR ≤ 0.06 as threshold values [68].

In order to verify whether the data fit the Rasch model [34], we first examined the monotonicity, checking whether the thresholds related to each item were correctly ordered. A threshold corresponds to the transition point between two different scores. At the transition point, the probability to obtain both scores is equivalent. When there are problems concerning the structure of a score, thresholds could be non-ordered. This indicates that one item does not function adequately, i.e., that its category scores are not logically ordered. We expected that increases in subjective well-being corresponded to increases in the scores, and that this pattern increased systematically according to a logic progression. We represented the order of the thresholds through the person-item map. Second, we checked the absence of local independence. Local independence indicates that the responses of a participant to one item of the scale do not influence the responses to other items of the scale for the same participant. Third, we examined the unidimensionality through the Martin-Löf test [69]. Then, we calculated the DIF, using the standardized P-DIF statistic [70], which allows for identifying uniform DIF without requiring an item response model approach. In addition, to examine the consistency of the responses, we considered the reliability in terms of the PSI. Finally, we checked the fit between the data and the Rasch model through the Andersen’s likelihood ratio test [71]. We then assessed the item performance considering the residual item fit statistics, expected to approximate to the normal distribution (with the mean close to 0 and the standard deviation close to 1). We considered two aspects of fit statistics: infit (i.e., mean square inlier-sensitive fit) and outfit (i.e., mean square outlier-sensitive fit). Infit and outfit mean-squares higher than 1.3 indicate underfit to the Rasch model, i.e., that the data are less predictable compared to what is predicted by the model; mean-squares lower than 0.7 indicate overfit to the Rasch model, i.e., that the data are more predictable compared to what is predicted by the model. Finally, based on the location of both items and persons, we applied the formula which enables to transform the raw global score into an interval logit scale [54]. We specify that, for conducting a Rasch analysis, the minimum number of participants is equal to 25 multiplied for the number of response categories plus one [55]. In our case, we used five (plus one) categories, and therefore the minimum number was 150. Therefore, the sample size resulted adequate. In the following analyses, we used the Rasch scores.

As regards the second aim, we calculated the correlations and the descriptive statistics concerning the SRW scale, the SWQ, achievement emotions, and grades in native language and mathematics. Following Cohen [72], we considered correlations comprised between 0.10 and 0.30 as small; between 0.30 and 0.50 as moderate; and higher than 0.50 as large. In relation to the third aim, we ran a linear mixed model (LMM), with class level (fourth-graders, seventh-graders) and gender (males, females) as categorical fixed effects, the school institute (four groups) as a random effect, and SRW (logit score) as the dependent variable. We performed a type III analysis of variance table with Satterthwaite’s method. For post-hoc tests we used the Bonferroni correction [73]. The level of significance was *p* < 0.05. Finally, for the fourth aim, we conducted another LMM with SRW (logit score) as the continuous fixed effect, subject-matter (native language, mathematics) as the categorical fixed effect, the school institute (four groups) as a random effect, and grades as dependent variables.

## 3. Results

### 3.1. Internal Validity of the SRW

#### 3.1.1. Exploratory and Confirmatory Factor Analyses

**Figure 1:**
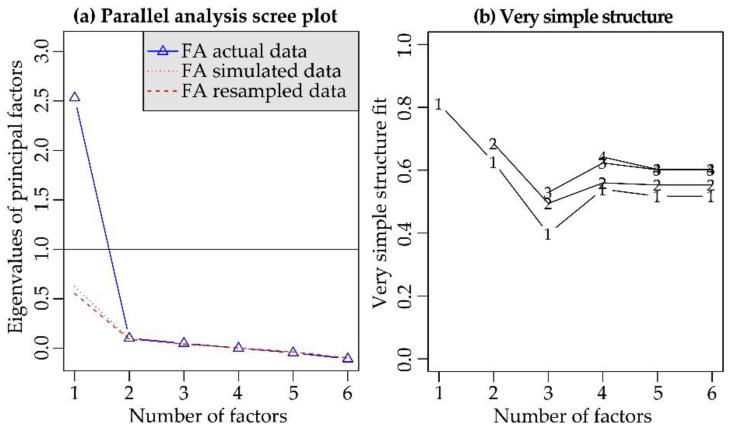
(**a**) Parallel analysis scree plot related to the SRW scale. The marked slope change between the first and second principal component shows that the first component is the most relevant. The red lines represent the random eigenvalues generated with the parallel analysis. The blue triangles represent the eigenvalues of each potential principal factor. Given that only the first eigenvalue was larger than the random eigenvalues, the analysis indicated that we should retain only one factor. (**b**) The very simple structure criterion indicated the optimal number of factors, i.e., one factor.

Bartlett’s test of sphericity suggested that there was a sufficiently significant correlation in the data for factor analysis, *χ^2^*(15) = 726.93, *p* < 0.001. The KMO measure of sampling adequacy indicated that the data seemed appropriate for factor analysis (KMO = 0.85). Both the parallel analysis and the very simple structure analysis revealed that the scale was unidimensional. We show in Figure 1a the parallel analysis scree plot and in Figure 1b the very simple structure.

We then conducted an EFA with oblimin rotation, which produced a unidimensional solution explaining 43% of the total variance. The fit indexes indicated that the model was adequate, TLI = 0.979, RMSEA = 0.048, and SRMR = 0.030. We reported in Table 1 the factor loadings concerning the six items. The mean correlation of the regression scores with the factor was 0.91. The multiple *R^2^* of the regression scores was 0.83. The minimum correlation of the possible factor scores was 0.66. The McDonald’s omega reliability index was 0.802, suggesting that the scale had a good reliability.Finally, we ran a CFA with one factor, which showed good fit indexes: CFI = 0.983, TLI = 0.972, RMSEA = 0.050, and SRMR = 0.028. Therefore, the analysis enabled to identify one factor for the SRW scale.

#### 3.1.2. Rasch Analysis

**Figure 2:**
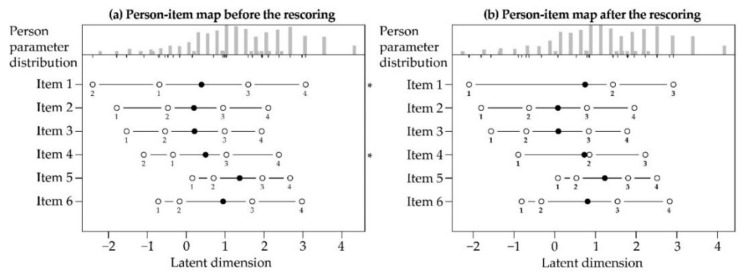
Person-item map relating to the six items of the SRW: (**a**) before the rescoring; (**b**) after the rescoring. The person-item map shows the relation between the location of persons’ well-being and items’ discriminatory capacities, represented along the same latent dimension. The person parameter (i.e., well-being) varies from lower scores on the left to higher scores on the right. The solid circles (•) represent the locations of items’ discriminatory capacities and the hollow circles (◦) represent the thresholds. The asterisks (*) represent the items with non-ordered thresholds.

We conducted the Rasch analysis through the partial credit model [74]. We first considered the monotonicity, examining the person-item map. As represented in Figure 2a, there was not monotonicity in the responses, given that the scores pertaining to items 1 and 4 did not have ordered thresholds. Therefore, we rescored items 1 and 4 to account for violations of the monotonicity assumptions. Specifically, the response scale for the two items changed from 1, 2, 3, 4, 5 to 1, 1, 2, 3, 4. We re-analyzed the monotonicity, which resulted confirmed, as indicated in Figure 2b.

**Figure 3:**
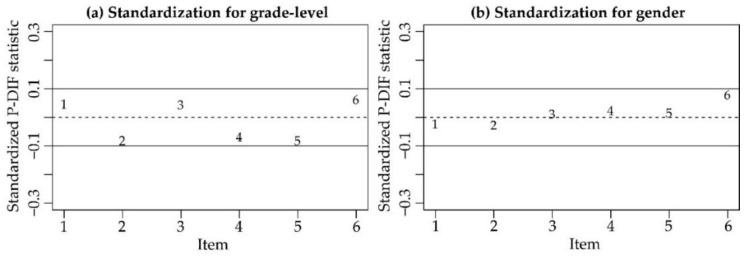
Representation of the standardized P-DIF statistic for (a) grade-level and (b) gender.

Then, we calculated the correlations between item residuals (−0.32 *< r* < 0.03), which gave no evidence of local dependence (i.e., there were not positive correlations larger than 0.30). Subsequently, through the Martin-Löf test, *Χ^2^*(119) = 88.485, *p* = 0.984, we confirmed that the SRW scale was unidimensional. Given that the values of the standardized P-DIF statistic, calculated separately for grade-level and gender, were comprised between −0.10 and 0.10 (Figure 3), we can state that the scale functioned in the same way for fourth-graders and seventh-graders, and for males and females.

The analysis of reliability for the total sample showed a PSI value equal to 0.80, resulting adequate. Finally, the Andersen’s likelihood ratio test, *Χ^2^*(21) = 18.224, *p* = 0.635, indicated that our data fit the Rasch model. In Table 2 we reported the goodness-of-fit of each item examined through the analysis of infit and outfit statistics: All the indexes were within the required range, indicating that the data were predicted by the model.

At this point, we were legitimized to sum the raw scores of the items in order to obtain a global score and to transform the SRW into an interval logit scale [75]. To favor comprehensibility, we transformed the range of the logit score as comprised between 1 and 10 (the total raw score was comprised between 6 and 28). It is worth noting that, to apply this formula, items 1 and 4 must be rescored as described above. We reported in Table 3 the conversion table including the raw scores and the corresponding logit scores.

### 3.2. External Validity of the SRW

#### Intercorrelations

In Table 4 we report the intercorrelations between the SRW (logit score) and the SWQ, achievement emotions, and grades. We also reported the descriptive statistics and the omega values for the SWQ and achievement emotions. First, students’ evaluations of the SRW were positively correlated with the three positive dimensions of the SWQ, while they were negatively correlated with the three negative dimensions, supporting *Hypothesis 2a*. In particular, correlations with positive attitudes towards school and enjoyment in school were large; correlations with positive academic self-concept and worries in school were moderate; and correlations with physical complaints in school and social problems in school were small. Second, for both settings, students’ evaluations of the SRW scale were moderately correlated with achievement emotions, positively with positive emotions and negatively with negative emotions, corroborating *Hypothesis 2b*. Finally, the SRW scale correlated positively and moderately with grades in both native language and mathematics.

### 3.3. Grade-Level and Gender Differences

#### Linear Mixed Models

**Figure 4:**
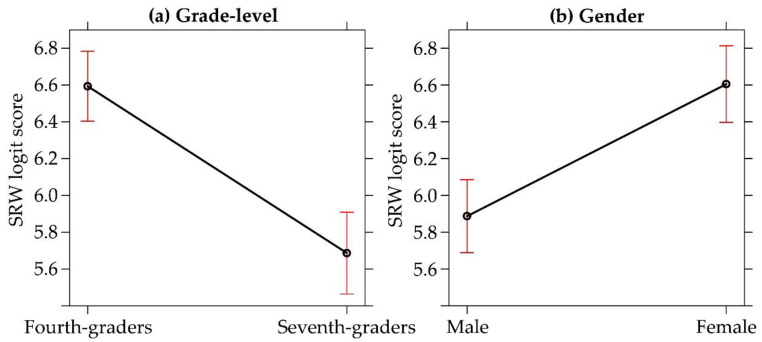
SRW logit score according to (**a**) grade-level and (**b**) gender. The bars represent the 95% CI.

The LMM revealed significant effects of grade-level, *F*(1, 341.20) = 47.934, *p* < 0.001, *η^2^_p_* = 0.12, and gender, *F*(1, 415.48) = 32.167, *p* < 0.001, *η^2^_p_* = 0.07. The interaction did not result significant (Figure 4). Well-being scores were higher for fourth-graders (*M* = 6.58, *SD* = 1.45, 95% CI [6.40, 6.76]) compared to seventh-graders (*M* = 5.73, *SD* = 1.17, 95% CI [5.56, 5.91]). In addition, they were higher for females (*M* = 6.59, *SD* = 1.37, 95% CI [6.39, 6.78]) compared to males (*M* = 5.93, *SD* = 1.36, 95% CI [5.75, 6.11]).

### 3.4. Relations Between Students’ Well-Being and Achievement

#### Linear Mixed Model

**Figure 5:**
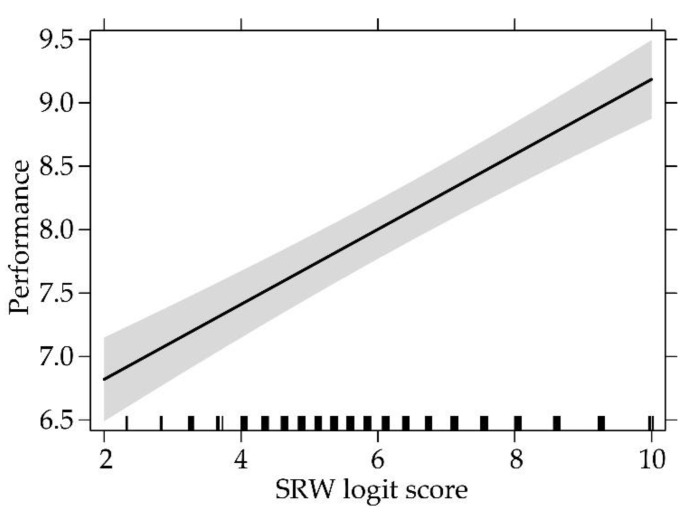
Influence of SRW logit score on performance. The band represents the 95% CI.

The LMM indicated a significant effect of the SRW logit score, *F*(1, 822.67) = 110.517, *p* < 0.001, *η^2^_p_* = 0.12, but not of the subject-matter nor of the interaction, on performance. In particular, as SRW logit score increased, also performance did the same (Figure 5).

## 4. Discussion

Through the adaptation to the Italian context of a brief scale deputed to measure well-being at school, the SRW, and its transformation using the Rasch analysis, our study aimed at extending current knowledge on how this key construct varies in the transition from primary to lower secondary school.

First, our analyses confirmed the goodness of the SRW scale as a brief unidimensional scale permitting to measure well-being at school, supporting *Hypothesis 1a*. Applying the Rasch model, we transformed the scale into an interval level measure [36,38]. This represents a strength of this work, because usually this approach is used within the fields of physical and natural sciences, and only more rarely within the psychological field. From a methodological perspective, our findings give the possibility to use the SRW taking advantage of the strengths of the Rasch model. In addition, we found that the SRW functioned equally for the two different age groups and across gender, confirming *Hypothesis 1b*. We highlight that we used the SRW scale with fourth and seventh-graders, in line with previous studies [5]. In the future, this instrument could be used to assess well-being with other age ranges, such as younger students.

Second, our findings supported the external validity of the SRW. The positive and significant correlations between the SRW and the six dimensions of well-being at school as measured by the SWQ [10] gave evidence of its construct validity, supporting *Hypothesis 2a*. Such result is particularly relevant taking into account that the SRW is a unidimensional measure, while the SWQ considers a variety of positive and negative factors related to well-being, which connote it as a multi-dimensional instrument. It is worth noting that the strongest correlations concerned the dimensions of the SWQ which pertain to the “feeling well” nuance of well-being [7], i.e., positive attitudes and enjoyment. The moderate correlations regarded another emotional dimension, worries, in this case negatively related to well-being, and academic concept, considered by theoretical approaches such as the control-value theory as one of the main antecedents of emotional states [20,21]. The weakest correlations concerned two dimensions that could be conceived as negative antecedents of well-being, i.e., physical complaints and social problems. At an applied level, we could speculate that when designing a research aiming at assessing well-being at school, a cautious analysis on the strengths and limitations of the two instruments should be made in order to choose the one that fits better with the contextual and organizational constraints, such as time for the administration, resources for the analyses, interest on a global measure rather than knowledge on specific nuances of well-being. As regards the relation between the SRW scale and achievement emotions, our findings extended previous results [5,13,32], supporting the discriminatory validity of the scale. As expected, correlations with pleasant emotions were positive, while correlations with unpleasant emotions were negative, confirming *Hypothesis 2b*.

Third, our analyses revealed a general decrease of school-related well-being as age increased, with lower scores for seventh-graders compared to fourth-graders, corroborating *Hypothesis 3a*. This confirmed a maladaptive trend typical of motivational and emotional constructs [16,17,18,19,22]. Moreover, females reported a higher school-related well-being compared to males. Our findings helped to disambiguate the complex patterns of factors underlying variations in school well-being, with a specific focus on class level and gender differences. In line with suggestions deriving from the perspective of positive psychology, future interventions and educational policies should monitor students’ well-being using reliable and valid instruments such as the SRW, and then promote it especially for those groups of students for which there is a deployment of well-being, in order to improve their quality of life [4].

Finally, our findings indicated that school-related well-being was positively associated to achievement for both native language and mathematics, confirming *Hypothesis 4a*. We highlight that this finding is based on the analysis of longitudinal data, with well-being measured before grades. It is important to note that school achievement is related to a wide range of individual cognitive (i.e., intelligence), motivational, emotional, and personality factors on the one hand, and to environmental factors such as socio-economic status, quality of instruction, and parental value attributed to instruction on the other hand [30]. The examination of these factors is clearly beyond the scope of this study. Nonetheless, the present findings are indicative of the predictive validity of the SRW scale, as its scores significantly predict achievement scores. In other terms, the more students feel well at school, the more they achieve. This result is of key relevance for its implications for school intervention strategies. Promoting well-being at school should be a major point of attention for educational policies not only because nowadays it is largely recognized as one of the essential objectives of the educational systems [1,2,3], along with traditional objectives focused on learning, but also because it is strictly connected to achievement itself.

The transition from primary to secondary education is a pivotal step associated with a number of developmental changes pertaining to the biological, cognitive, emotional, and social domain [e.g., 1,14]. Only recently attention has been paid to how well-being varies in this transition [e.g., 1,15]. However, there is still a paucity of brief instruments deputed to measure well-being at school. The SRW scale has the great advantage to be very short, and therefore it can be administered easily. This is particularly advantageous taking into account some constraints typical of surveys with children (e.g., low attention, necessity to gather data on well-being together with other measures in long surveys, etc.). Therefore, we adapted to the Italian context the SRW scale, taking advantage from using the Rasch model to adapt and develop an instrument which respects the properties of the fundamental measurement [34,35,36].

This study suffers from some limitations. First, the SRW scale could be quite a simple scale for secondary school students. Nevertheless, previous studies have already used it with students of a similar age [5]. Second, our design was in part cross-sectional. School-related well-being and achievement emotions were assessed at a single point in time, and therefore this does not allow to draw conclusions about causal relations between these variables; future studies might use fully longitudinal designs to overcome this limitation. Third, well-being and achievement emotions referred to school in general, while achievement regarded specific domains. Nevertheless, native language and mathematics are the main subject-matters for the class levels that we took into account. Future studies should explore further these relations using the same level of generality for all constructs. Fourth, we did not examine the impact of technology on students’ well-being during the transition, similarly to many studies published recently [1]. The role of technology within learning environments is rapidly increasing, also due to the radical changes in learning strategies related for example to the current COVID-19 pandemic. Therefore, future research should explore how well-being is linked to technology-related phenomena characterizing learning contexts, such as the achievement emotions felt during digital learning activities [65]. In addition, we did not investigate further variables that play a central role in the school, such as interpersonal relationships with peers or academic stress. Further studies should explore the influence of these constructs. Fifth, due to time restrictions and to avoid the risk of fatigue related to a long questionnaire, the study examined only a limited set of variables that might be related to well-being at school (i.e., achievement in native language and mathematics). Apart from achievement, other variables could be influenced by well-being at school, such as cognitive and affective adaptability and misconduct [5].

## 5. Conclusions

Our study enabled to assess well-being at school for fourth-graders and seventh-graders, by adapting and validating the Italian version of the SRW scale taking advantage of the strengths of the Rasch model. From a theoretical perspective, our data contributed to clarify the role of class level and gender as key factors which influence well-being at school, extending previous literature; in addition, they confirmed the relevance of students’ well-being for their performance. From an applied perspective, our findings highlight the need for developing interventions to support students in the transition from primary to secondary school, which is such a pivotal time in their learning path.

## Figures and Tables

**Table 1:** Item description in the English and Italian versions and factor loadings for the SRW scale.

Item number	English Version	Italian Version	Loadings
1	School is going well for me.	A scuola mi va tutto bene.	0.59
2	I feel good at school.	Io sto bene a scuola.	0.77
3	School fulfills my needs.	La scuola mi offre quello di cui ho bisogno.	0.46
4	I feel comfortable at school.	Mi sento bene a scuola.	0.73
5	I like going to school.	Mi piace andare a scuola.	0.64
6	All in all, I am content with my day-to-day school experiences.	Quando ripenso alla mia giornata a scuola, sono contento/a.	0.66

**Table 2:** Infit and outfit mean square statistics (MSQ) of each item of the SRW scale.

Item Number	Infit-MSQ	Outfit-MSQ
1	0.89	0.90
2	0.71	0.76
3	1.12	1.20
4	0.75	0.75
5	0.86	0.96
6	0.81	0.81

**Table 3:** Conversion table from raw scores of the items of the SRW scale to logit scores.

Raw Scores	Logit Scores
6	1.000
7	1.689
8	2.293
9	2.822
10	3.284
11	3.687
12	4.040
13	4.352
14	4.632
15	4.887
16	5.128
17	5.362
18	5.598
19	5.846
20	6.113
21	6.408
22	6.741
23	7.119
24	7.552
25	8.047
26	8.615
27	9.263
28	10.000

**Table 4:** Correlations, number of items, means (*M*), standard deviations (*SD*), and omega values for the SRW scale, the SWQ, achievement emotions, and grades in native language and mathematics.

Variable	1	2	3	4	5	6	7	8	9	10	11
1. SRW	-										
2. SWQ - Positive attitudes towards school	0.78 ***	-									
3. SWQ - Enjoyment in school	0.57 ***	0.55 ***	-								
4. SWQ - Positive academic self-concept	0.50 ***	0.40 ***	0.38 ***	-							
5. SWQ - Worries in school	−0.31 ***	−0.27 ***	−0.25 ***	−0.26 ***	-						
6. SWQ - Physical complaints in school	−0.14 ***	−0.13 ***	−0.06	−0.18 ***	0.44 ***	-					
7. SWQ - Social problems in school	−0.21 ***	−0.13 ***	0.02	−0.12 *	0.27 ***	0.37 ***	-				
8. Positive emotions	0.42 ***	0.38 ***	0.52 ***	0.31 ***	−0.24 ***	−0.08	−0.04	-			
9. Negative emotions	−0.33 ***	−0.33 ***	−0.23 ***	−0.25 ***	0.41 ***	0.50 ***	0.47 ***	−0.18 ***	-		
10. Grades - Native language	0.37 ***	0.35 ***	0.34 ***	0.39 ***	−0.30 ***	−0.15 ***	−0.04	0.28 ***	−0.21 ***		
11. Grades - Mathematics	0.31 ***	0.30 ***	0.25 ***	0.40 ***	−0.33 ***	−0.22 ***	−0.09 *	0.22 ***	−0.25 ***	0.79 ***	
Number of items	6	3	3	3	3	4	3	10	10	1	1
*M*	6.23	3.68	3.57	3.77	2.88	2.11	2.10	3.23	2.03	8.00	8.06
*SD*	1.40	0.82	0.80	0.74	1.03	0.93	0.94	0.83	6.36	1.14	1.29
Omega	-	0.62	0.65	0.66	0.70	0.68	0.78	0.75	0.77	--	--

*** *p* < 0.001.
